# A dataset of human decision-making in teamwork management

**DOI:** 10.1038/sdata.2016.127

**Published:** 2017-01-17

**Authors:** Han Yu, Zhiqi Shen, Chunyan Miao, Cyril Leung, Yiqiang Chen, Simon Fauvel, Jun Lin, Lizhen Cui, Zhengxiang Pan, Qiang Yang

**Affiliations:** 1 Joint NTU-UBC Research Centre of Excellence in Active Living for the Elderly (LILY), Nanyang Technological University, Singapore 639798, Singapore; 2 School of Computer Science and Engineering, Nanyang Technological University, Singapore 639798, Singapore; 3 Department of Electrical and Computer Engineering, The University of British Columbia, Vancouver, British Columbia Canada V6T 1Z4; 4 Institute of Computing Technology, Chinese Academy of Sciences, Beijing 100190, China; 5 School of Computer Science and Technology, Shandong University, Jinan, Shandong 250101, China; 6 Department of Computer Science and Engineering, Hong Kong University of Science and Technology, Hong Kong, China

**Keywords:** Computational science, Decision making

## Abstract

Today, most endeavours require teamwork by people with diverse skills and characteristics. In managing teamwork, decisions are often made under uncertainty and resource constraints. The strategies and the effectiveness of the strategies different people adopt to manage teamwork under different situations have not yet been fully explored, partially due to a lack of detailed large-scale data. In this paper, we describe a multi-faceted large-scale dataset to bridge this gap. It is derived from a game simulating complex project management processes. It presents the participants with different conditions in terms of team members’ capabilities and task characteristics for them to exhibit their decision-making strategies. The dataset contains detailed data reflecting the decision situations, decision strategies, decision outcomes, and the emotional responses of 1,144 participants from diverse backgrounds. To our knowledge, this is the first dataset simultaneously covering these four facets of decision-making. With repeated measurements, the dataset may help establish baseline variability of decision-making in teamwork management, leading to more realistic decision theoretic models and more effective decision support approaches.

## Background & Summary

The complex social, economic, engineering, and scientific activities of today’s world often require effective teamwork if successful outcomes are to be achieved. A team usually consists of members with diverse capabilities who collaborate, often under the leadership of a team manager, towards a common purpose. In order for a team to produce desired outcomes, the collective resources of the team (e.g., the team members’ time and productivity) must be managed effectively. The effectiveness of teamwork management decisions depends on the complex interactions between team members’ capabilities and characteristics, the team manager’s decision-making strategies, and the nature of the tasks to be undertaken by the team.

Decision-making is one of the fundamental cognitive processes of human beings. It involves making rational, heuristic, or intuitive choices in activities of daily living as well as in complex scientific, economic, and management problems^1^. When managing teamwork, decision-making is often characterized by uncertainty and resource constraints^2^. An example is software project management. A team of software engineers, each with different skills and characteristics, need to work together to deliver a software system on time with good quality. The manager, who needs to decide how to allocate tasks of different complexities among the team members over many iterations during the project, often faces uncertainty about a particular team member’s performance. Team members, being human, face resource constraints as they each have different limits in terms of productivity. The manager may have to update his/her perception about team members’ capabilities iteratively over the course of the project by observing their performance in order to make subsequent decisions.

In the above scenario, multiple decision theoretic approaches (e.g., Choice under Uncertainty^3^, Inter-temporal Choice^4^, and Complex Decisions^5^) are involved. Under such situations, which can be broadly characterized as *decision-making under uncertainty and resource constraints*, decisions may not always result in desirable outcomes^6^. One of the most damaging outcomes is the phenomenon of *herding*. Herding refers to situations in which a large number of requests concentrate on a small number of resources perceived to be of high quality, causing these resources to be overloaded while leaving other resources unused^7,8^. It can lead to cascading failures where resources are depleted one after the other, eventually resulting in system breakdown^9^. In the aforementioned software engineering team management example, herding can occur when the team manager overly relies on a few reliable team members, which may result in fatigue and reduced team morale.

Much existing research in decision-making has been based on theoretical constructs^10^ which often reflect the researchers’ decision framework rather than those of the people actually involved in a given system. How different people manage teamwork under different situations and the effectiveness of their strategies are not well understood due to a lack of large-scale detailed data. Without a sound understanding of people’s teamwork management decision-making behaviours under uncertainty and resource constraints, the effectiveness of decision support tools may also be hampered. Therefore, it is important to collect data about people’s teamwork management decisions in order to facilitate further research in this field.

Interactive games offer a promising means to engage research participants for large-scale collection of detailed data. This approach allows research participants to be exposed to different scenarios and enables traditional research tools such as surveys to be administered remotely. Moreover, the participants’ detailed decision-making behaviour trajectory data can be tracked unobtrusively without interrupting their game play experience^11^. We hypothesize that more frequent quantitative tracking of people’s decision-making behaviour trajectory over time under various decision situations may lead to a better understanding of emergent behaviours.

In December 2013, we launched the *Agile Manager* game platform (http://agilemanager.algorithmic-crowdsourcing.com/). The game puts participants into various scenarios of managing a team of virtual worker agents (WAs) with diverse characteristics. It unobtrusively collects participants’ sequential decision-making behaviour trajectory data over time under various conditions of uncertainty and resource constraints. The dataset enables researchers to explore the processes of decision-making under such conditions over time with information regarding:

1. The decision situations (including information about the behaviour characteristics of the WAs and their workload at any point in time during the game sessions),
2. The participants’ decisions strategies (including both their self-reported strategies and the actual individual decisions made at different points in time during the game sessions),
3. The decision outcomes (including both the participants’ actual scores, a breakdown of the scores lost due to different reasons, and comparison with a computational decision support approach^12^), and
4. The participants’ emotional responses to the decision outcomes.

To the best of our knowledge, this is the first public dataset simultaneously covering these four facets of the decision-making process. This multi-faceted open decision dataset from real users fulfills a need in decision-making research^13^, and will be of great interest to researchers.

The publicly available dataset described in this paper was collected through crowdsourcing^14^ during the period from December 2013 to October 2016 from 1,144 participants aged between 18 and 89 with various educational and cultural backgrounds. All study procedures were conducted in English. It may help establish baseline variability of decision-making under uncertainty and resource constraints. This unique dataset aims to accelerate the research leading to more realistic, evidence-based models and effective decision support approaches.

## Methods

### Participant enrollment

The Agile Manager game platform^15^ was made available starting in December 2013 through a dedicated website hosted by the Joint NTU-UBC Research Centre of Excellence in Active Living for the Elderly (LILY), Nanyang Technological University (NTU), Singapore for personal computers running the Windows XP operating system or higher. Enrollment is open to anyone who chooses to download and play the game. With the game downloaded and installed, a prospective participant self-navigates through the registration process. The aims of the study, the participants’ rights, and the data collection policies are explained in an electronically rendered informed consent form which a prospective participant must agree to in order to join the study and play the game. Ethical oversight of this research was provided by the Research Advisory Committee of the LILY Research Centre.

### Study design

The Agile Manager game simulates a team of ten worker agents (WAs), each with different capabilities in terms of quality of work and productivity. A participant acts as the project manager and decides how to allocate a given set of tasks to the WAs over multiple iterations, while information about each WA’s quality of work and productivity is gradually revealed to the participant.

#### Game session

Once enrolled, a participant is given a choice of six game levels. The settings for the six game levels (Game Levels.xlsx, Data Citation 1) are shown in Table 1. These settings govern the total workload a participant needs to allocate among the WAs as well as the behaviour patterns of the WAs so as to create various scenarios. The relationship between the productivity and quality of work of a WA is controlled by the *Speed versus Quality Trade-off* (SvQ) variable. If SvQ is set to −1, WAs which produce high quality results have small task processing capacities. If SvQ is set to 1, WAs which produce high quality results have large task processing capacities.

#### Tasks

A total of 30 tasks are defined in the game (Tasks.xlsx, Data Citation 1). For game levels in which only 20 tasks are required, the tasks from the dataset are sorted in descending order of their values and the top 20 tasks are used. The variables pertaining to a task are listed in Table 2. Tasks with higher difficulty generally require more effort and carry higher values (i.e., rewards). However, tasks with IDs 12 and 13 are designed to be of high difficulty but require low effort and carry low values to introduce a degree of variation into the decision-making process.

#### Worker agents

There are a total of 20 WAs, each programmed to exhibit different characteristics in terms of quality and speed of work (Worker Agents.xlsx, Data Citation 1). As WAs have limited productivity, they are potential bottlenecks (overburdened resources) in the scenarios simulated by this game. Each WA has a pending task queue that can hold an unlimited number of tasks. A WA works on the tasks in this queue on a first-come-first-served (FCFS) basis. The variables associated with a WA are listed in Table 3.

#### The game play

**Figure 1:**
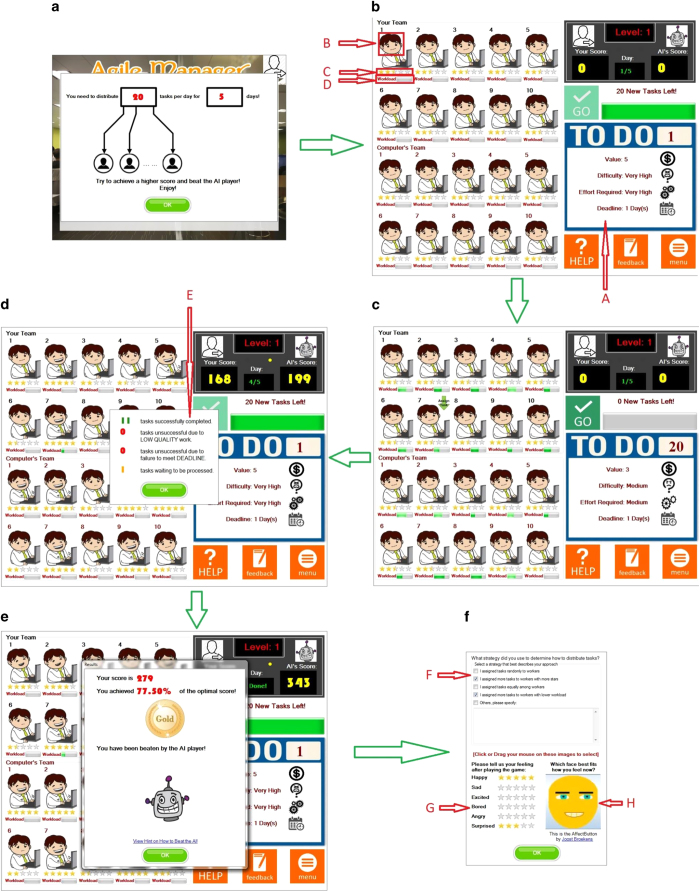
The user interface of the game at different stages of the game play.

In each game session, a participant selects a game level and acts out his/her task allocation strategy by assigning tasks to WAs, while the game platform unobtrusively collects data associated with each decision. In each game level, the participant faces a different condition as defined by the interaction between the behaviour pattern of the WAs and the number and nature of the tasks that need to be completed. Figure 1 shows a typical sequence of a game play in the Agile Manager platform. The objective for a participant is to obtain the highest score possible from tasks successfully completed by deciding how tasks should be assigned to the WAs in each round of the game (Fig. 1a). In order to be deemed successful, a task must be completed with high quality and before the stipulated deadline. The participant’s score is increased by the value of the task if both the quality and timeliness requirements are fulfilled. Otherwise, his/her score remains unchanged.

A total of 10 WAs are under a participant’s control during a given game session. In game levels 1, 3, and 5 in which the SvQ variable is set to 1, WAs with ID numbers 1 to 10 are used. In game levels 2, 4, and 6 in which the SvQ variable is set to −1, WAs with ID numbers 11 to 20 are used. At the start of a round, the tasks are presented to a participant one by one with value, difficulty, required effort, and deadline information shown in a summary format (Area A in Fig. 1b). A participant is required to assign the task to one of the ten WAs in his/her team.

The participant only has partial information regarding the behaviour patterns of the WAs, as would be the case in real-world situations. The performance of each WA is tracked by the game platform using two variables: (1) its reputation; and (2) its current workload. The reputation of a WA is computed following the approach discussed in ref. 16. The calculated reputation value for a WA can be interpreted as the probability of a task being successfully completed by the WA (i.e., with high quality and before its deadline) based on information available up to the current round. At the beginning of each game session, all WAs display a neutral reputation of 0.5. The participant learns the reputation of each WA through interactions (i.e., task assignments). It is displayed in the form of the emotional expression of a WA (Area B in Fig. 1b) and a star rating (Area C in Fig. 1b). The higher the reputation value, the more stars, and the happier the expression of the WA. Although the reputation value can take on many values in [0,1], the game divides the value range of [0,1] uniformly into 10 discrete levels and displays it as the star ratings and facial expressions of a WA. The current workload is displayed as the percentage of the *Max Productivity* of a WA discounted by multiplying it with the *Average Worker Agent Productivity Output Rate* (Area D in Fig. 1b). If a WA is overloaded, the current workload bar does not exceed the 100% marker. The participant can view a detailed breakdown of key statistics for each WA by pressing on the WA’s star rating (Area E in Fig. 1d). These statistics include:

- The number of tasks successfully completed by the WA;
- The number of failed tasks due to low quality;
- The number of failed tasks due to missed deadlines;
- The number of tasks waiting to be processed by the WA.

Once all pending tasks have been assigned to WAs, the participant can press the ‘Go’ button to initiate the simulation of the current round (denoted as ‘Day’ in the user interface) to allow the WAs to work on the tasks. At the start of the next ‘Day’ (round) in the game, the outcomes from the previous round are reflected in both the participant’s score and the updated star ratings for the WAs (Fig. 1d).

The result of a task completed by a WA is either *high quality* or *low quality*. The *High Quality Output Probability* variable of a WA *i* is denoted as *Pr* (*i*). The probability of a high quality result for task *τ*, *q*(*τ*), is modelled as:

(1)q(τ)=1[Pr(i)≥d(τ)]

where *d*(*τ*) is the difficulty of task *τ*. 1_[condition]_ is an indicator function which evaluates to 1 (i.e., high quality result) if [condition] is true; otherwise, it evaluates to 0 (i.e., low quality result).

In order to provide a benchmark for participants to know how well their strategies perform, which hopefully motivates them to improve their strategies, an artificial intelligence (AI) competitor is included in the game. It follows the task allocation strategy proposed in ref. 12 to control an identical team of WAs and competes with the participant during each game session. Basically, the AI approach adopts a combination of greedy reputation-based approach with load-balancing considerations.

At the end of each game session, the overall outcome of a participant’s decisions made during the session (information about the score and whether the participant beat the AI competitor) is presented to him/her (Fig. 1e). The participant is then required to report the strategy he/she used during the game session. He/she can choose a mixture of available descriptive options and/or provide textual descriptions (Area F in Fig. 1f). In addition, the participant is also required to report his/her emotions after knowing the outcome of the game session. The participant can specify the degrees of the six basic emotions^17^ (Area G in Fig. 1f), and drag his/her mouse across the *Affectbutton*^18^ to select an emoticon that best represents his/her facial expression at the moment (Area H in Fig. 1f).

A video walkthrough of how a participant plays the game can be found at https://youtu.be/PjungGYmr9E. Participants could choose to exit from a game session at any time.

#### Game Sessions

**Figure 2:**
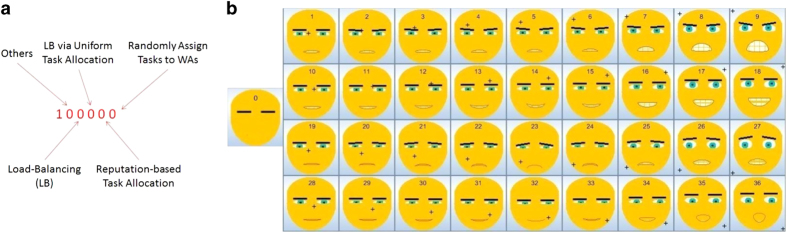
Participants’ self-reports. (**a**) The meaning of each bit in the *User Strategy Index* variable; (**b**) the *AffectButton* emoticons^18^ corresponding to the *Facial Expression ID* variable values.

Each completed game session is entered into the database with a record containing the key information (shown in Table 4) regarding the collective outcome of the participant’s decisions (Game Sessions.xlsx, Data Citation 1). The *User Strategy Index* variable is represented as a binary number which allows participants to express pure or combination strategies they used during a game session. The meaning of each bit is shown in Fig. 2a. For example, a *User Strategy Index* of 100,010 means that the participant reports allocating tasks to WAs purely based on their star ratings (i.e., WAs with more stars receive more tasks). A *User Strategy Index* of 101,010 means that the participant also takes into account the WAs’ current workload while trying to allocate more tasks to WAs with more stars. The leftmost bit of the *User Strategy Index* variable is always set to 1 so that in case the participant does not want to report his/her strategy, the *User Strategy Index* variable indicates 100,000.

Two aspects of the participant’s self-reported emotional responses to the game session outcome are recorded: (1) facial expression, and (2) a mixture of the six basic emotions. As the game does not assume that the participants have access to or are willing to use web cameras, an interactive widget—the *AffectButton*^18^—is incorporated into the game for the purpose of capturing facial expressions. A participant is asked to drag the mouse across the AffectButton to select the expression he/she thinks best fits his/her current feelings. The result is captured as an integer index number in the range of [0,36] (with 0 being the default value, i.e., a neutral facial expression). The facial expression corresponding to each index number can be looked up in Fig. 2b. The ‘+’ sign on each emoticon indicates the mouse position that triggers the corresponding emoticon to be selected. An 11-point Likert scale^19^ is used to capture each of the six basic emotions reported by the participants (with 0 indicating the lowest intensity and 10 indicating the highest intensity).

The *Start Time* and the *End Time* can be used to calculate how long it takes a participant to complete a given game session.

#### Participants’ decisions

Within each game session, a participant may allocate between 100–300 tasks to WAs depending on which game level he/she was playing (20 or 30 tasks for 5 or 10 rounds). At the end of each round, a snapshot of the conditions of all the WAs under the participant’s management is taken. A record for each WA is created at this point in time in the Decisions dataset (Decisions.xlsx, Data Citation 1). The record consists of the fields listed in Table 5. A WA’s reputation and productivity forms the context information a participant can observe before making his/her decisions. By comparing each WA’s backlog queues across different rounds under one game session, together with information about the WAs’ productivity, it is possible to build a time series representing the changes in each WA’s workload and, thus, infer how the participant allocated tasks among the WAs in response to what he/she observed during the game session.

### Data collection and distribution

The game platform recorded all data collected for this study through interactions with a remote MySQL database server hosted by goddady.com. Participants’ task allocation decisions, the context information surrounding these decisions (i.e., WAs’ reputation, current workload, task difficulty, task value), and the effects of these decisions (i.e., the participant’s score, the AI participant’s score, the scores lost due to low quality results/missed deadlines by the participant and the AI participant, emotional responses by the participant to the results) are transmitted to the database server via an Internet connection in real time.

Anonymized study data, consisting of data reflecting participants’ decision context, decision-making behaviours, emotional responses to the decision outcomes as well as their self-reported categorizations and descriptions of their strategies, are exported to Synapse for distribution to researchers. Synapse (https://www.synapse.org/) is a general-purpose data sharing service where members can share and analyze data collaboratively under a governance framework. It is developed and operated by Sage Bionetworks. Many scientific datasets collected using similar approaches to ours, such as the *mPower* dataset^20^, adopt Synapse for data storage and distribution.

### Code availability

The dataset is released under the Open Database License (ODbL) and is publicly available at (Data Citation 1).

The Agile Manager game software used to build the dataset can be found at (Download the Agile Manager Game, Data Citation 1). Readers can make use of this tool to conduct their own empirical studies. Nevertheless, as all decision-making behaviour data are sent to a dedicated server for storage, readers are advised to contact the authors of this paper to acquire unique organization codes prior to the commencement of their studies so that data from their own studies can be easily identified and exported. In case that the original authors of this paper are not in a position to be contacted or respond, readers can direct such requests to the Secretariat of the Association for Crowd Science and Engineering (ACE) at secretariat@crowdscience.org.

## Data Records

**Figure 3:**
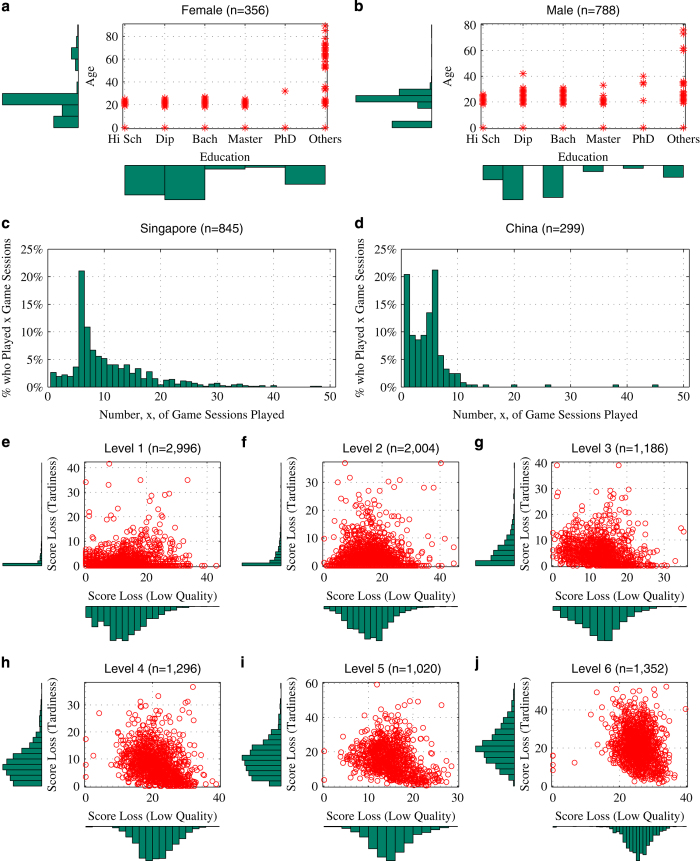
An overview of the dataset. Sub-figures (**a**,**b**) show the scatter-plots and the density distributions of the age and education levels for female and male participants, respectively. Sub-figures (**c**,**d**) show the density distributions of the number of game sessions by participants from Singapore and China, respectively. Sub-figures (**e**–**j**) illustrate the scatter-plots and the density distributions of the normalized scores (in the range of 0–100%) lost by the participants due to 1) low quality of work and 2) failure to meet deadlines in game levels 1–6, respectively. The higher the game level, the higher the overall workload placed on the virtual team of WAs (i.e., the more challenging for decision-making).

A total of 1,144 participants consented to the study and agreed for their data to be used for research purposes. Their de-identified demographic information is stored in the Users dataset (Users.xlsx, Data Citation 1). Table 6 shows the categories of participants’ demographic information released in this dataset. Among them, 356 are female and 788 are male. The distribution of age and highest level of education among the female and male participants are illustrated in Fig. 3a,b, respectively. The short forms ‘Hi Sch’, ‘Dip’, and ‘Bach’ are used to denote ‘High School’, ‘Diploma’, and ‘Bachelor's Degree’, respectively. In terms of geographic distribution, 845 participants are located in Singapore while 299 are in China. Participants have the option not to disclose their age information. If they choose to do so, their age in the dataset is set to 0. A total of 256 participants chose not to disclose their age.

The participants’ personality and affective-oriented disposition information has been collected during the account registration stage. The personality survey questions, PQ1 to PQ10 in Table 6, are from the Ten-Item Personality Inventory (TIPI)^21^. Possible values for PQ1 to PQ10 are: 1-‘Disagree strongly’, 2-‘Disagree’, 3-‘Neither agree nor disagree’, 4-‘Agree’, and 5-‘Agree strongly’. The affective-oriented disposition survey questions, AQ1 to AQ20 in Table 6, are from the Positive and Negative Affect Schedule (PANAS)^22^ Section E. Possible values for AQ1 to AQ20 are: 1-‘Never’, 2-‘Rarely’, 3-‘Sometimes’, 4-‘Usually’, and 5-‘Always’. Participants have the option not to take part in the personality survey or the affective-oriented disposition survey. The personality and affect questionnaire data are only available on a subset of participants. In total, 151 participants took part in both surveys, 10 participants took part in only the TIPI survey, and another 10 participants took part in the PANAS survey. If a participant did not take part in either or both of these two surveys, the corresponding columns in the Users dataset remain empty. More detailed information on the two surveys can be found in the *Readme.pdf* file associated with the dataset (Readme.pdf, Data Citation 1).

The distribution of the number of game sessions played by participants from Singapore and China are shown in Fig. 3c,d, respectively. Due to the nature of the study, follow up is nonuniform across individual participants. Overall, 2.65% of the participants from Singapore completed only one game session, while 20.41% of the participants from China completed only one game session. On average, a participant from Singapore completed 10.9 game sessions, while a participant from China completed 4.9 game sessions.

Figure 3e–j show the distributions of the scores lost by the participants due to low quality work and late completion as a result of their task allocation strategies in different game levels. The number of times each game level has been played by the participants is also indicated in the titles of these figures. In total, the participants completed 9,854 game sessions and generated 495,533 decision trajectory records in the Decisions dataset (Decisions.xlsx, Data Citation 1).

All datasets and the game platform are stored and accessible via the Synapse platform in a public project with associated metadata and documentation (https://www.synapse.org/#!Synapse:syn5909526/files/).

## Technical Validation

The data provided herein are obtained using standard game-based data capturing techniques^23^. The data collection actions are triggered by pre-defined in-game events in the Agile Manager game system. These events are triggered by mouse clicks on specific regions of the game interface (e.g., button press after users filled up required forms). The events rely on the underlying keyboard and mouse hardware, operating systems software and the network connectivity. The game supports Windows XP and higher versions of Microsoft operating systems. As the data collection relies on mature technologies, it is highly unlikely that the data collected would have been corrupted during this phase.

For game interfaces which involve users typing information into forms, we have implemented standard data validation mechanisms^23^. These include:

1. Given names and surnames cannot contain numbers;
2. Date of birth is selected from a software calendar interface rather than being typed in to avoid date format discrepancies;
3. All ‘signs in any text field are replaced with’ signs to prevent MySQL database from misinterpreting them as part of the query syntax;
4. Email addresses should contain ‘@’ and at least one’. ‘after the ‘@’ character;
5. Education and gender are selected from dropdown lists so as to standardize the terminology;
6. Each participant must agree to the terms of the study before being allowed to create an account;
7. At the end of each game session, at least one task allocation strategy options must be selected before the game can proceed;
8. If a participant selects ‘Others, please specify’ from the task allocation strategy options, the strategy description textbox must not be empty;
9. During each round of a game session, all tasks must be allocated to the WAs before the game is allowed to proceed;
10. To ensure that task allocation decision data are received by the remote server, at the end of each round a game session, the game waits until it receives the acknowledgement signal from the server before proceeding to the next round. Otherwise, the game session is automatically marked as ‘unfinished’ in the database;
11. If a participant terminates the game before a game session is finished, the game session is also automatically marked as ‘unfinished’ in the database.

Before publishing the dataset, we conducted a round of manual data cleaning. Firstly, all unfinished game session records (6,077 in total) were excluded from the *Game Sessions* table. The *Decisions* table was also cleaned so that records generated from the unfinished game sessions (147,916 in total) were excluded. Secondly, all finished game sessions with no self-reported task allocation strategy (125 in total) were excluded from the dataset. This situation can occur if the game crashes or is forcibly terminated while a participant is reporting his task allocation strategies. The above operations were executed using MySQL queries. Finally, the *User Strategy Description* texts were removed from game session records in the dataset if the texts are illegible. Examples of illegible texts include ‘xvg’, ‘vsdvds’, and ‘dfgre’, etc. A total of 111 such descriptions were removed, leaving 259 legible user strategy descriptions in the current dataset.

## Usage Notes

Due to the novel nature of the dataset released in this paper, governance structures have been put in place to ensure the proper dissemination of the data. A researcher who is interested in accessing the dataset must complete the following steps:

1. Become a registered user of *Synapse* (https://synapse.org);
2. Validate the Synapse user account following guidelines from the Synapse Access and Compliance Team (ACT).

As the dataset is disseminated by Synapse, it imposes overarching ‘Conditions for Use’ (https://www.synapse.org/) which must be followed by potential dataset users.

### Implications

To the best of our knowledge, the rich and large-scale dataset reported in this paper is the first detailed study of human decision-making behaviours under uncertainty and resource constraints. Participants who contributed to the dataset come from diverse backgrounds in terms of age, gender, education, and geographic locations. New dimensions of information including the contextual information prior to the decisions, the detailed sequence of decisions over multiple iterations, the impact of these decisions on the decision goal fulfillment, and the participants’ self-reported emotional responses to the decision outcomes and explanations about their decision strategies will stimulate researchers to discover new insights into how different people behave under different decision situations.

The dataset has the potential to shed light on important and challenging research questions in social science. For instance:

#### How effective are the different task allocation strategies under different conditions?

As each game level represents a unique setting in terms of overall workload level and worker agent behaviour characteristics, this information can be jointly queried with the participants’ self-reported strategies and the outcomes they have achieved for each game session in the *Game Sessions* table to study the effectiveness of various strategies under different conditions. Primary variables concerning the outcome of a participant’s decisions during a game session include the normalized score for the session, the score lost due to tasks completed with low quality, and the score lost due to missing the deadline.

Based on the primary variables, an important secondary variable—the score lost due to tasks which are completed both with low quality and past the deadline for a given game session *α*, *S*_*l,t*_(*α*)—can be computed as follows:

(2)Sl,t(α)={0,ifSl(α)+St(α)+S(α)≤Smax(α)Sl(α)+St(α)+S(α)−Smax(α),otherwise

where *S*_*l*_(*α*) is the score lost due to low quality task results in game session *α*; *S*_*t*_(*α*) is the score lost due to tasks completed past their deadline in game session *α*; *S*(*α*) is the total score achieved for game session *α*; and *S*_max_(*α*) is the maximum achievable score for game session *α*. This secondary variable can help researchers gain further insights into conditions under which different strategies can result in the most serious form of failures—tasks which are completed with low quality while also missing the deadline.

By answering this question, researchers can quantify the effects of various strategies under different workload and worker behaviour conditions in order to guide the formulation of more effective decision support mechanisms in the future.

#### What are the preferred task allocation strategies by people from different backgrounds under different conditions?

To study this problem, a researcher can jointly query the participants’ demographic information, the settings information of each game level, and the self-reported strategies by the participants for each of these levels. The relationships among this combination of variables are likely to be non-linear. Thus, we foresee neural network techniques^24^ to be useful in this case. These results may help researchers understand the task allocation strategy preferences by people from different backgrounds, and design decision support mechanisms which are more persuasive to different sub-populations.

#### What are the variations in executing the same self-reported task allocation strategies by people from different backgrounds under different conditions?

With individual task allocation decisions captured by our dataset, researchers can investigate one step beyond the second research question to gain deeper understanding in individual variations when executing the same self-reported task allocation strategies. To do so, researchers can leverage the workload backlog data for each WA in different rounds under each game session. The *Decisions* table contains a WA’s current workload (including the IDs of the tasks in its backlog queue, the total workload as measured by the number of tasks in its backlog queue, and the total workload as measured in terms of effort units). The actual task allocation decisions made by a participant at the beginning of each round of game is reflected by the IDs of the tasks in the WAs’ backlog queue.

By sorting the table contents by Session ID, by Round and then by Worker Agent ID, the average backlog workload assigned to each WA can be computed by summing the Worker Agent Backlog values over all Rounds under a Session ID, and then divided by the number of Rounds under that Session ID. Since the table also contains WAs’ reputation values at each round of a game session, researchers can use this information to study how WAs’ reputations influence people’s task allocation decisions under different self-reported task allocation strategies. Researchers can potentially utilize this information from the dataset to construct Markov Decision Processes (MDPs)^25^ in order to build more realistic models of human decision-making under various conditions.

#### Can a participant’s demographic information and game play outcomes predict their emotional response after playing?

As the dataset contains the participants’ demographic information, their scores and their AI opponent’s scores, as well as the corresponding self-reported intensities of the six basic emotions after each game session, it is possible to study the relationships between these factors. If a researcher wishes to study more specific scenarios related to task allocation decisions in which the scores lost due to low quality or tardiness also need to be taken into account, he could do so by including these two variables from the *Game Session* table. As the relationships are also likely to be non-linear, neural networks can be promising techniques for this purpose.

The results may help researchers understand more general game play scenarios in which a participant competes with game AI, and make situation-aware personalized emotional interactions or interventions in such games a possibility. Preliminary results studying participants’ strategies and composite emotional responses based on the dataset have yielded promising new findings. In ref. 26, it has been found that the participants’ characteristics and their decision outcomes are significantly correlated to their emotional responses.

Our dataset holds the potential to lead to new modelling approaches, particularly in the research domains of Choice under Uncertainty, Inter-temporal Choice, and Complex Decisions, and to improve the effectiveness of decision support systems in teamwork management.

## Additional Information

**How to cite this article**: Yu, H. *et al.* A dataset of human decision-making in teamwork management. *Sci. Data* 4:160127 doi: 10.1038/sdata.2016.127 (2017).

**Publisher**’**s note**: Springer Nature remains neutral with regard to jurisdictional claims in published maps and institutional affiliations.

## Supplementary Material



## Figures and Tables

**Table 1:** Game Level Settings Data.

**Variable Name**	**Range**	**Description**
Level	1–6	The unique identification number of a game level
Speed versus Quality Trade-off (SvQ)	{−1, 1}	The correlation between the *High Quality Output Probability* and the *Max Productivity* of a WA
No. of Rounds	{5, 10}	The number of iterations within each game level, during which a participant is required to allocate tasks to WAs
Tasks per Round	{20, 30}	The number of tasks a participant is required to allocate to WAs during each round of a game
Average Worker Agent Productivity Output Rate	11–20	The actual discounted *Max Productivity* a WA can output during each round of a game

**Table 2:** Task Settings Data.

**Variable Name**	**Range**	**Description**
ID	1–30	The unique identification number of a task
Value	1–5	The score a participant receives if the task is completed successfully by the assigned WA
Difficulty	{0.2, 0.4, 0.6, 0.8, 1.0}	The difficulty value of the tasks (with 1 being the hardest)
Effort Required	1–5	The workload placed on a WA by this task (expressed in terms of Effort Units)
Deadline	1	The number of rounds in the game by which the task must be completed

**Table 3:** Worker Agent Settings Data.

**Variable Name**	**Range**	**Description**
ID	1–20	The unique identification number of a WA
High Quality Output Probability	{0.1, 0.2,..., 1}	The probability of the WA completing a given task with high quality
Max Productivity	11–20	The maximum workload a WA can complete per round of a game (expressed in terms of Effort Units)
SvQ Setting	{−1, 1}	Each game session is associated with its own SvQ setting value. This variable determines under which game session the WA should be included.

**Table 4:** Game Session Data.

**Variable Name**	**Range**	**Description**
ID	NA	The unique identification number of a game session
User ID	NA	The unique identification number of the participant who played this game session
Game Level	1–6	The identification number of the game level played in this game session
Player Score	0–100%	The score obtained by the participant in this game session
Player Score Loss (Low Quality)	0–100%	The score lost by the participant as a result of tasks being completed with low quality in this game session
Player Score Loss (Tardiness)	0–100%	The score lost by the participant as a result of tasks not completed before their stipulated deadlines in this game session
AI Score	0–100%	The score obtained by the AI participant in this game session
AI Score Loss (Low Quality)	0–100%	The score lost by the AI participant as a result of tasks being completed with low quality in this game session
AI Score Loss (Tardiness)	0–100%	The score lost by the AI participant as a result of tasks not completed before their stipulated deadlines in this game session
User Strategy Index	‘100,000’–‘111,111’	The index value expressing the participant’s self-reported task allocation strategy used in this game session
User Strategy Description	NA	The participant’s explanation about his/her task allocation strategy used in this game session (optional)
Facial Expression ID	0–36	The unique identification of the emoticon selected by a participant to represent his/her emotion
Happiness	0–10	The participant’s self-reported degree of happiness
Sadness	0–10	The participant’s self-reported degree of sadness
Excitement	0–10	The participant’s self-reported degree of excitement
Boredom	0–10	The participant’s self-reported degree of boredom
Anger	0–10	The participant’s self-reported degree of anger
Surprise	0–10	The participant’s self-reported degree of surprise
Start Time	NA	The date and time the game session started
End Time	NA	The date and time the game session ended

**Table 5:** Participants’ Decision Data.

**Variable Name**	**Range**	**Description**
ID	NA	The unique identification number of a WA’s current situation
Session ID	NA	The unique identification number of the game session during which this snapshot was taken
Round	1–10	The unique identification number of the game round within this game session during which this snapshot was taken
Worker Agent ID	1–20	The ID of the WA
Worker Agent Backlog (No. of Tasks)	≥0	The WA’s current workload after the participant has finished allocating all tasks in this round (measured in terms of number of tasks)
Worker Agent Backlog (No. of Effort Units)	≥0	The WA’s current workload after the participant has finished allocating all tasks in this round (measured in terms of Effort Units)
The Backlog Queue	NA	The IDs of the pending tasks for a WA, separated by semi-colons
Worker Agent Reputation	0–1	The current reputation of the WA

**Table 6:** Participants’ Information.

**Variable Name**	**Range**	**Description**
ID	NA	The participant’s unique identification number
Gender	‘Male’, ‘Female’	The participant’s gender
Education	‘High School’, ‘Diploma’, ‘Bachelor’, ‘Master’, ‘PhD’, ‘Others’	The participant’s highest level of education
Country	‘Singapore’, ‘China’	The country the participant is located in
Age	NA`	The participant’s age at the time when he/she joined the study
Account Creation Time	NA	The exact date and time a participant joined the study
PQ1—PQ10	{1,2,3,4,5}	10 survey questions used for assessing the participant’s personality
AQ1—AQ20	{1,2,3,4,5}	20 survey questions used for assessing the participant’s affective-oriented disposition
